# Symposium on evacuation and shelter-in-place strategies in nuclear disasters: preventing disaster-related deaths

**DOI:** 10.1093/jrr/rrag010

**Published:** 2026-07-17

**Authors:** Chihiro Matsumoto, Toyoaki Sawano, Michio Murakami, Shogo Takahara, Kaori Muto, Hiroyuki Beniya, Masaharu Tsubokura

**Affiliations:** Department of Radiation Health Management, Fukushima Medical University School of Medicine, 1 Hikariga-oka, Fukushima City, Fukushima 960-1295, Japan; Department of Radiation Health Management, Fukushima Medical University School of Medicine, 1 Hikariga-oka, Fukushima City, Fukushima 960-1295, Japan; Department of Surgery, Jyoban Hospital of Tokiwa Foundation, 57 Kaminodai, Jyoban Kamiyunagayamachi, Iwaki City, Fukushima 972-8322, Japan; Research Center for Community Health, Minamisoma Municipal General Hospital, 2 Chome-54-6 Haramachiku Takamicho, Minamisoma City, Fukushima 975-0033, Japan; Department of Health Risk Communication, Fukushima Medical University School of Medicine, 1 Hikariga-oka, Fukushima City 960-1295, Fukushima, Japan; Center for Infectious Disease Education and Research, The University of Osaka, 1-10 Yamadaoka, Suita, Osaka 565-0871, Japan; Risk Analysis Research Group, Nuclear Safety Research Center, Japan Atomic Energy Agency, Shirakata 2-4, Tokai-mura, Naka-gun, Ibaraki 319-1195, Japan; Human Genome Center, The Institute of Medical Science, The University of Tokyo, 4-6-1 Shirokane-dai, Minato-ku, 108-8639, Tokyo, Japan; Laboratory of Biomedical Ethics and Co-design (BECoD), RIKEN Center for Integrative Medical Sciences, 1-7-22 Suehiro-cho, Tsurumi-ku, Yokohama City, Kanagawa 230-0045; Orange Medical and Social Services, 1 Chome-2-20 Tawara, Fukui, 910-0018, Japan; Department of Radiation Health Management, Fukushima Medical University School of Medicine, 1 Hikariga-oka, Fukushima City, Fukushima 960-1295, Japan

**Keywords:** nuclear disaster, disaster prevention, evacuation, radiation exposure

## Abstract

On 27 September 2024, at the 67th Annual Meeting of the Japan Radiation Research Society at the Kitakyushu International Conference Center in Kokura City, the program organizers held a symposium titled ‘Symposium on Evacuation and Shelter-in-place Strategies in Nuclear Disasters: Preventing Disaster-Related Deaths.’ This symposium aimed to examine practical disaster-prevention strategies at the operational level in disaster-affected areas during nuclear disasters and featured presentations and general discussions with four speakers. The topics included evacuation during the Great East Japan Earthquake and Fukushima Daiichi nuclear power plant accident; ethical problems regarding medical policy during the coronavirus disease 2019 pandemic; on-site issues in supporting areas affected by the Noto Peninsula earthquake; and estimates of the effect of reducing radiation exposure by shelter-in-place in four scenarios during a nuclear disaster. The discussion provided insights into decision-making processes during nuclear disasters. The symposium concluded that government must collaborate with medical and nursing care professionals to develop specific disaster-prevention measures. These plans should be tailored to the particular circumstances of the disaster and the unique conditions of each facility.

## INTRODUCTION

The disaster-prevention efforts of Japan, including those for nuclear accidents, have evolved from major catastrophes, such as the Isewan Typhoon, Great Hanshin-Awaji Earthquake, JCO criticality accident and Fukushima Daiichi nuclear power plant (FDNPP) accident. The JCO criticality accident occurred in a conversion factory at the JCO tokai site. The accident required approximately 19 hours to stop criticality. Consequently, 3 individuals were exposed to serious radiation doses, including 2 individuals were exposed to fatal radiation doses. In this accident, the government issued a shelter-in-place order for residents living with a 10-km radius of the JCO tokai site [1]. The FDNPP accident occurred in 2011, triggered by the Great East Japan Earthquake (GEJE), at the facility located on the coastal region of Fukushima Prefecture. Following the seismic and tsunami events, radiation readings in the vicinity of the facility surged to a maximum of over 400 μSv/h, because of the damage to reactor buildings. The accident resulted in the widespread dispersal of radioactive materials into the environment; consequently, an evacuation order was issued for areas within a 20-km radius of the plant, while an instruction for shelter-in-place was issued for a 20–30 km radius [2]. Furthermore, areas outside the 20-km radius where additional annual cumulative doses exceeded 20 mSv were designed as deliberate evacuation areas [3]. Following the 1999 JCO criticality accident and 2011 FDNPP accidents, the ‘Special Measures Act on Nuclear Emergency Preparedness’ and ‘Guidelines for Nuclear Disaster Preparedness’ were implemented. These guidelines established and updated international standard-based response policies include evacuation, shelter-in-place, and zone-based resident protection (precautionary action zone, urgent protective action planning zone [UPZ]) [4, 5].

Despite the establishment of nuclear disaster-preparedness plans, significant on-site response challenges have persisted, including communication and awareness gaps among officials, healthcare staff, and residents, as well as inadequacies in human resources, flexible decision-making, and medical expertise, even with improved material preparedness [6, 7]. When planning future nuclear-disaster responses, lessons from other disasters are informative. For example, depending on circumstances, emergency evacuation may be necessary during a nuclear disaster; however, it can increase the risk of death among individuals vulnerable to disasters, irrespective of disaster type [8]. Additionally, the importance of identifying shared challenges was highlighted based on communication challenges in the Chemical, Biological, Radiological, Nuclear, and Explosive context and the coronavirus disease 2019 (COVID-19) pandemic [9]. Nevertheless, it has been noted that a characteristic of nuclear disasters is that evacuations may be prolonged owing to widespread radioactive contamination [10]. Nuclear disaster countermeasures include elements shared with other disasters and elements unique to nuclear events. It is essential to study both the commonalities and differences, particularly with disasters triggered by natural hazards, to derive lessons.

On 27 September 2024, at the 67th Annual Meeting of the Japan Radiation Research Society at the Kitakyushu International Conference Center in Kokura City, a symposium titled ‘Symposium on Evacuation and Shelter-in-place Strategies in Nuclear Disasters: Preventing Disaster-Related Deaths’ was held to examine the realities and challenges of nuclear disaster response from various perspectives. The symposium title was modified from the original title, ‘Evacuation and Shelter-in-Place During Nuclear Emergencies: To Prevent Disaster-Related Death,’ for this manuscript. This symposium brought together a doctor, an ethicist, a home doctor, and a researcher to discuss nuclear disaster response. They shared insights on evacuation-related deaths in Fukushima, ethical issues in emergency response to COVID-19 older adult care, older adult support, evacuation during the Noto Peninsula earthquake, and the quantitative effectiveness of shelter-in-place in reducing radiation exposure. Their diverse perspectives highlighted real-world challenges in evacuation, shelter-in-place, and disaster-related deaths, aiming to inform future nuclear disaster-prevention strategies.

## PROGRAM OF THE SYMPOSIUM

### Overview and lessons learned on disaster-related deaths in Minamisoma City, Fukushima prefecture, after the FDNPP accident

(Toyoaki Sawano, Department of Surgery, Jyoban Hospital of Tokiwa Foundation, Fukushima, Japan)

In a nuclear disaster, prolonged evacuation may be necessary because of radioactive contamination, thus preventing disaster-related deaths. A disaster-related death, as defined by the Disaster Condolence Grant Payment Law, refers to a death caused by a worsening injury or illness from the physical toll of evacuation. It is certified by a local committee and is a legal, not medical, term. Certification issues include the lack of uniform standards, requirements for bereaved family applications, and instances of certification without medical evaluation. For citizens, complex application documents and uncertainty regarding eligible cases are problematic, highlighting the need for consultation desks.

The 2011 GEJE and FDNPP accident led to the widespread release of radioactive materials and issuance of evacuation orders within 20 km of the FDNPP. All hospitals in affected areas had to evacuate their patients and staff. Previously, no plans existed for such a large-scale release of radioactive materials. Despite staff efforts, 43 patients died immediately after evacuation from the three hospitals, with 17 more deaths within 3 months. Furthermore, reports demonstrate an increased risk of death among older adult nursing home residents in Minamisoma City who are forced to evacuate [11–14]. Notably, lessons from the FDNPP accident emergency evacuations highlight the need to address two types of fatalities: immediate and mid-to-long-term deaths related to evacuation. Evacuating large numbers of hospitalized patients, especially those with severe or bedridden conditions, has proven challenging owing to transportation difficulties and time constraints. Although not evacuating might seem plausible based solely on the radiation levels in a nuclear disaster, widespread damage to buildings and infrastructure in complex disasters often makes this impossible. Ultimately, decisions to evacuate hospitals and facilities during such events are influenced by several factors beyond radiation levels, unless contamination is extremely high [15]. When investigating disaster-related deaths in Minamisoma City, more than 30% of deaths were reported to have occurred more than 6 months after the earthquake [16]. Additionally, respiratory and circulatory diseases often cause disaster-related fatalities. Nevertheless, individuals not requiring extensive nursing care tend to die from malignant tumors in the later stages of the disaster. Therefore, acute-phase medical care should prioritize those needing high-level nursing, whereas chronic-phase medical examinations should be provided to those with lower care needs [17]. Disaster-related deaths often affect vulnerable individuals with preexisting conditions, such as disabilities, malignant tumors, or those subjected to frequent post-disaster relocation [16, 18]. To reduce disaster-related deaths in the future, collecting high-quality data to identify vulnerable groups is warranted, thereby requiring the expansion of medical and welfare follow-ups for health-vulnerable individuals, extending beyond the immediate aftermath to the medium- and long-term.

### Difficulties in caring for older adults in COVID-19 measures

(Kaori Muto, Human Genome Center, The Institute of Medical Science, The University of Tokyo, Tokyo, Japan; Team Director, Laboratory of Biomedical Ethics and Co-design (BECoD), RIKEN Center for Integrative Medical Sciences)

Nuclear disasters cause profound ethical dilemmas in medical care. During the FDNPP accident, hospitals within a 5-km radius were forced to evacuate, resulting in patient deaths due to the burden of evacuation, while hospitals 20–30 km away also struggled with staff and supply shortages. Importantly, hospitals are often confronted with profound ethical dilemmas as they engage in life-or-death triage decisions regarding evacuation, treatment, and resource allocation.

The Japanese experience with the COVID-19 pandemic highlights similar dilemmas. From a public health ethics perspective, measures restricting individual rights are permissible only if they are demonstrably effective and time-limited. Under Article 19 of the Infectious Diseases Act, prefectural governors may ‘recommend’ hospitalization for patients with Class I infectious diseases. Following the Omicron variant emergence, infections surged and overwhelmed hospitals. However, hospitalization, especially for high-risk older adults, remained strongly encouraged, significantly burdening the healthcare system. Notably, healthcare workers bear substantial responsibility for admission and discharge decisions, especially when the needs of the patients are unclear. Even short hospital stays often reduce the activities of daily living of older adult patients, leaving many unable to return to their original lives. Moreover, even after the government lifted its COVID-19 measures in April 2022, some hospitals and nursing care facilities continued to impose strict restrictions on visits and accompaniment, resulting in a decline in the quality of life among older adults.

Future nuclear disaster preparedness must address these ethical dilemmas through peacetime planning and training by prioritizing emergency transportation and lifesaving measures, avoiding harsh decision-making burdens on frontline responders, critically evaluating preventive evacuation strategies, and promoting advance care planning among older adults to clarify their values and wishes.

### Considering the balance between evacuation and living (from the Noto Peninsula earthquake experience)

(Hiroyuki Beniya, Orange Medical and Social Services, Fukui, Japan)

A nuclear disaster could lead to extensive radioactive contamination and prolonged evacuations, posing significant challenges for residents. Research on the GEJE and FDNPP accidents suggests that the health of evacuees was impacted by environmental changes, limited medical access in new locations, and social isolation. Addressing these support challenges in nuclear disaster planning is crucial, as further highlighted by the January 2024 Noto Peninsula earthquake. In the Oku-Noto region of Ishikawa Prefecture, a case study from the Gochamaru Clinic underscored difficulties in supporting welfare evacuation shelters. The Noto region, with a 48% aging rate, saw many older adults experience worsening frailty after the disaster. Of 22 designated welfare shelters in Wajima City, only one, namely the ‘Umi to Sora’ group home, was functional. Evacuation to gymnasiums often leads to strength loss and disorientation among older adults. Because maintaining a normal environment in Wajima was difficult, a wide-area welfare shelter was established in Fukui Prefecture. Providing roles and motivation for older disaster victims proved important, as they often became entirely reliant on caregivers. Disaster relief efforts highlighted key issues: extending normal medical and welfare support into disaster periods, leveraging the expertise of home-care staff, and maintaining normalcy during evacuations through tailored welfare-shelter management. Planning must also account for individuals who fall through the cracks of aid systems, whose numbers may grow. To prevent isolation, communities should foster social connections and conduct emergency simulations during peacetime.

### Radiation exposure doses to the inhabitants and effectiveness of sheltering in a nuclear emergency

(Shogo Takahara, Risk Analysis Research Group, Nuclear Safety Research Center, Japan Atomic Energy Agency, Ibaraki, Japan)

In Japan, sheltering is a protective action that reduces public radiation exposure during a nuclear emergency [5]. This action is taken to minimize radiation doses from three exposure pathways: internal exposure due to inhalation, external exposure from radioactive plumes (cloudshine), and deposited radionuclides (groundshine). Here, we evaluated the dose reduction effects of sheltering in various nuclear accident scenarios.

Dose reduction effects were evaluated using the dose reduction factor ($DRF$), which is defined by the following equation:


$$ {DRF}_{total}=\frac{\sum_i{DRF}_i\bullet{D}_{I,i}}{\sum_i{D}_{O,i}}, $$

where ${D}_{o,i}$ is the effective dose accumulated over 7 days post-release of radioactive materials. ${DRF}_i$ is the dose reduction factor for exposure pathway $i$, and its values depend on environmental (weather), social (house material, age, building density), and accident conditions (nuclide type, release timing/duration). ${DRF}_{total}$ due to sheltering was evaluated for four accident scenarios: (i) a large late release scenario, wherein a large-scale release of radioactive materials caused by pressure-induced reactor containment vessel failure [19, 20]; (ii) a controlled release scenario, wherein controlled containment venting is successfully implemented as an accident management measure to mitigate the consequences of an accident; (iii) Cs137 100TBq (Nuclear Regulation Authority of Japan [NRA] scenario), wherein the total amount of radioactive materials to the atmosphere when all radioactive materials were converted to Cs137 is 100TBq, which is a criterion for the Evaluation of the Effectiveness of Measures to Prevent the Spread of Hydrogen Explosions, by the NRA regarding previous severe accident research [21]; (iv) the 1F2 scenario, which is the estimated source term released from Unit 2 in the FDNPP accident [22]. Evaluations of doses and DRFs were performed assuming 2 days indoors followed by 5 days without further protective measures.

Consequently, the ${DRF}_{total}$ value can be divided into two types, depending on the radionuclides that mainly contributed to exposure. In the first case, iodine and cesium were the main contributors to the administered dose received by the people. Large late-release scenarios and 1F2 scenarios were included in this type. In these scenarios, exposure occurred from groundshine and inhalation, and the value of ${DRF}_{total}$ was approximately 0.4. Second, radiation from noble gases mainly contributed to the total received dose. In this type of situation, most exposure occurred from the cloud shine pathway through the roof. The materials of the roof were thinner than those of the wall, and the shielding effect was lower than that in the first case. Therefore, the DRF value was approximately 0.6. Controlled-release and NRA scenarios were included in this type of case.

Furthermore, we evaluated the radiation doses within a radius of 30 km from the release point. For the large-release scenario and the 1F2 scenario, the effective doses to people within a radius of 30 km may be above 100 mSv, which is adopted by the International Atomic Energy Agency as a criterion for implementing protective measures. Nonetheless, the controlled release and NRA scenarios are unlikely to exceed this criterion, even if protective measures are not undertaken, including sheltering. Additionally, in the protective strategy according to the NRA guideline, temporary relocation is implemented when the ambient dose equivalent rate exceeds 20 μSv/h in areas where sheltering is designated. Our evaluation results exhibited a very low possibility of areas exceeding 20 μSv/h within the UPZ for the NRA scenario.

## DISCUSSION

This general discussion focuses on disaster-related deaths resulting from the evacuation of medical and nursing care facilities during nuclear disasters. Disaster-related death prevention is a major issue during such catastrophes. A study comparing the deaths from the GEJE in Fukushima, Miyagi, and Iwate prefectures reported that the proportion of disaster-related deaths was the highest in Fukushima, where the nuclear accident had occurred, potentially because evacuations were prolonged due to widespread radioactive contamination from the nuclear accident [10]. Moreover, a study of a nursing care facility in Minamisoma City, Fukushima Prefecture, which was forced to evacuate because of the FDNPP accident, suggested that sudden evacuation actions increase the risk of death of older adults and other vulnerable disaster victims [13]. Furthermore, the characteristics and causes of death of people who die in disaster-related cases vary depending on the time since the disaster [16]. To prevent disaster-related deaths, understanding the full picture of such fatalities, that is, what situations and types of people are at risk of disaster-related deaths, and developing evacuation plans that reduce these risks, is warranted. Three major issues regarding disaster-related deaths associated with the evacuation of medical and nursing care facilities were presented.

First, data are insufficient to fully understand disaster-related deaths, owing to the diversity of circumstances and factors involved. Several studies have identified characteristics, namely diseases, medical history, and age, that predispose individuals to death and delineated the timing of death after a disaster [16, 17, 23, 24]. Nonetheless, to date, only data from Minamisoma City, one of the municipalities where disaster-related deaths occurred during the FDNPP accident, have been available. Furthermore, owing to the limitations in applying for disaster condolence money, the number of deaths recognized as disaster-related may be underestimated, thereby hindering disaster-related death data acquisition. Furthermore, owing to the unique nature of the disaster, generalizing the knowledge gained from the FDNPP accident to other disasters or future disasters remains challenging. A significant feature of the GEJE was the combination of a disaster triggered by natural hazards and a nuclear disaster; moreover, an excessive response to the nuclear event led to many disaster-related deaths owing to the physical and mental burdens of evacuation. To respond to disasters with varying degrees of impact from nuclear accidents and wind and flood damage, it is necessary to analyze diverse categories of related deaths.

Second, detailed standards regarding whether people should evacuate or take shelter-in-place in the event of a nuclear disaster are missing. Operational Intervention Levels 1 and 2 set out criteria for deciding whether to evacuate, shelter-in-place, or temporarily relocate based on the air dose rate. Nevertheless, standards addressing factors other than the air dose rate, such as the difficulty in maintaining hospital functions due to a shortage of workforce and supplies at medical and nursing care facilities, are lacking. Importantly, sudden evacuation actions at medical and nursing care facilities increase the health risks to those vulnerable to disasters. However, in reality, in the case of a complex disaster, there may be situations where it remaining in the facility is challenging because of damage to buildings and infrastructure [15, 25]. When the decision to evacuate or shelter-in-place is delayed, medical and nursing care facilities remain uncertain whether to prepare for evacuation or for sheltering, causing staff confusion and exhaustion [16]. For example, during the FDNPP accident, hospitals in areas under shelter-in-place orders were unable to continue shelter-in-place because of a lack of supplies and workforce and were forced to evacuate in an emergency, resulting in disaster-related deaths due to sudden evacuation actions [26]. The decision to evacuate or shelter-in-place should be made as early as possible; however, it depends on multiple factors, including the extent of radioactive material release, extent of facility damage, available manpower and supplies, status of external support, patient condition, and acceptable radiation-exposure levels for hospital staff and patients [15]. Going forward, it will be necessary to develop a method that integrates these factors to guide the choice between evacuation and shelter-in-place. One approach is to build a simulation that uses these factors as inputs and outputs predicted health risks for patients and staff at the facility.

Third, to save lives immediately after a disaster, multi-layered advance preparations are necessary, with cooperation among the government, medical and welfare organizations, and local levels during peacetime. First, the government must be responsible for formulating criteria for prioritizing transportation and lifesaving in emergencies and presenting them to medical institutions. Additionally, medical institutions should be trained in ethical judgments during peacetime to prepare for emergencies. Furthermore, to ensure that disaster victims are not isolated and can live as normally as possible, it is crucial to maintain local communities that include medical and welfare workers, as well as residents. To reduce the risk of disaster-related deaths in future nuclear disasters, governments and medical facilities must work closely to consider concrete disaster responses from a field perspective.

In conclusion, the authors shared and discussed issues regarding practical disaster-prevention measures at the operational level in disaster-affected areas in the event of a nuclear disaster. In the event of a future nuclear disaster, it is essential for the government and medical and nursing care staff to collaborate on implementing specific disaster-prevention measures tailored to the circumstances of the disaster and the condition of each facility.
